# *ADGRG6* Promotes Pancreatic Adenocarcinoma Progression Through the NF-κB/STAT6 Axis and Modulation of the Tumor Immune Microenvironment

**DOI:** 10.3390/cimb47120991

**Published:** 2025-11-27

**Authors:** Lisha Li, Zhen Yu, Xuehua Lu, Pei Yang, Shuxia Zhang, Qinghua Lin, Junyong Han

**Affiliations:** Fujian Key Laboratory of Medical Analysis, Fujian Academy of Medical Sciences, Fuzhou 350001, China; lisa_faoms@fjms.ac.cn (L.L.); yuzhen@fjms.ac.cn (Z.Y.); lxh6675@fjms.ac.cn (X.L.); ypfjyyzz@fjms.ac.cn (P.Y.); zoengjoyce@fjms.ac.cn (S.Z.); campass123@163.com (Q.L.)

**Keywords:** *ADGRG6*, *GPR126*, pancreatic adenocarcinoma, NF-κB, STAT6, GATA3, tumor immune microenvironment, 3D culture, zebrafish xenograft, biomarker

## Abstract

Background: Adhesion G protein-coupled receptor G6 (*ADGRG6*), also known as *GPR126*, has been implicated in several malignancies. However, its expression pattern, clinical significance, and mechanistic role in pancreatic adenocarcinoma (PAAD) remain unclear. Methods: We combined multi-omics analyses, tissue microarray immunohistochemistry, and a series of functional experiments, including 2D and 3D spheroid cultures, zebrafish xenografts, and murine tumor models—to investigate the expression, clinical significance, and mechanism of *ADGRG6* in PAAD. The association between *ADGRG6* expression and immune infiltration was assessed using TIMER and GEPIA databases, followed by mechanistic validation through *ADGRG6* modulation in PAAD cell lines. Results: *ADGRG6* was significantly overexpressed in PAAD and correlated with larger tumor size, higher grade, advanced TNM stage, and poor overall survival. Multivariate logistic regression confirmed that high *ADGRG6* expression was independently associated with higher pathological grade. Functionally, *ADGRG6* silencing markedly inhibited PAAD cell proliferation, migration, and invasion in both 2D and 3D cultures, as well as in zebrafish and nude mouse xenograft models. Integrated transcriptomic and immune analyses revealed that *ADGRG6* expression positively correlated with mast cells, macrophages (M1/M2), Th2/Th17 subsets, and interferon–responsive neutrophils. Mechanistically, *ADGRG6* silencing reduced STAT6 phosphorylation and GATA3 expression, consistent with the suppression of the NF-κB→STAT6→GATA3 axis. Conclusions: *ADGRG6* functions as an oncogenic driver in PAAD, promoting tumor progression and fostering an immunosuppressive microenvironment via NF-κB/STAT6 signaling. These findings not only broaden the mechanistic understanding of ADGRG6 function but also suggest it as a promising target for therapeutic intervention in PAAD.

## 1. Introduction

Pancreatic cancer remains one of the most lethal malignancies, with a 5-year relative survival rate of approximately 13% [1]. Although the survival rate of patients with pancreatic cancer has been improving, the rate of its enhancement has been slower compared with other cancers [2]. PAAD, a highly malignant and rapidly progressing tumor, shares similar characteristics with other aggressive cancers such as ovarian cancer [1,3]. It presents significant challenges for early diagnosis and effective treatment, and its clinical manifestations are often nonspecific in the early stages. PAAD originates from the pancreatic exocrine ducts and constitutes the majority of pancreatic cancer cases [4]. Obesity, smoking, chronic pancreatitis and late-onset diabetes are several risk factors for pancreatic cancer [5,6]. Unfortunately, most patients are diagnosed at advanced stages, where treatment options become limited, and survival rates remain poor.

Despite advances in therapeutic strategies, the molecular mechanisms underlying PAAD’s initiation and progression remain incompletely understood. PAAD is known to progress through precursor lesions, primarily pancreatic intraepithelial neoplasia (PanIN) and intraductal papillary mucinous neoplasm (IPMN), whose genetic evolution involves recurrent driver mutations such as *KRAS*, *TP53*, *CDKN2A*, and *SMAD4* [7,8,9]. However, recent single-cell and spatial multi-omic studies have revealed profound intertumoral heterogeneity and complex evolutionary trajectories that challenge this classical linear model [10]. Moreover, selective pressures from the tumor microenvironment, along with emerging evidence for epigenetic reprogramming, further complicate the landscape of PAAD progression and therapeutic resistance [9,11]. Consequently, while the general framework of PAAD genetic evolution is established, the key molecular mechanisms that sustain its progression and immune evasion remain unresolved. In particular, how tumor cells interact with and remodel their microenvironment through specific signaling mediators is still poorly defined.

Adhesion G-protein-coupled receptor (adhesion GPCR) represents an important subfamily of the GPCR superfamily [12]. These receptors are unique in that they mediate signaling through interactions with extracellular matrix components, influencing cellular adhesion, migration, and differentiation. The contemporary nomenclature of adhesion GPCRs (with the previous names featured in parentheses) is as follows: Adhesion GPCR D1 (*ADGRD1*) (*gpr133*), *ADGRF1* (*gpr110*), *ADGRG6* (*gpr126*), and others. Previous studies have highlighted the critical roles of adhesion GPCRs in tumor biology. For instance, *ADGRD1* (*gpr133*) is highly expressed in glioblastoma and plays a role in tumor progression [13]. *ADGRF1* acts as an oncogene in lung and prostate cancers and serves as a prognostic biomarker in osteosarcoma [14,15], while *ADGRG1* and *ADGRL4* exert tumor-suppressive functions in melanoma and retinoblastoma, respectively [16,17]. Furthermore, adhesion GPCRs have been linked to immune cell infiltration and modulation of the tumor microenvironment (TME), suggesting their potential as therapeutic and immunomodulatory targets [18].

*ADGRG6* (*gpr126*), an orphan receptor within this subfamily, plays essential roles in Schwann cell myelination development and disease regulation [19,20]. Recent studies have implicated *ADGRG6* in tumor progression and immune regulation, suggesting it as a promising candidate for cancer diagnostics and therapy [21,22]. Notably, Wu et al. identified *ADGRG6* as a novel prognostic biomarker for PAAD that promotes tumor progression by stabilizing mutant p53 and activating the EGPR/NF-κB signaling axis [23]. While their work revealed a tumor cell-intrinsic oncogenic mechanism, the immunological functions of *ADGRG6* and its broader impact on the TME remain poorly defined.

Building upon these findings, the present study aimed to systematically elucidate the oncogenic and immunoregulatory roles of *ADGRG6* in PAAD. Through integrative bioinformatics analyses, tissue microarray validation, and functional assays in vitro and in vivo, including 2D and 3D cell cultures, zebrafish xenografts, and murine tumor models, we investigated its expression pattern, prognostic impact, and underlying molecular mechanisms.

We hypothesized that *ADGRG6* is overexpressed in PAAD and predicts unfavorable clinical outcomes by promoting tumor cell proliferation and migration through NF-κB/STAT6-mediated signaling and modulation of the TME. The primary endpoints of this study were (i) the association between ADGRG6 expression and patient prognosis and (ii) the functional and mechanistic evidence linking ADGRG6 to tumor progression and immune regulation in PAAD.

## 2. Materials and Methods

### 2.1. Tissue Microarray and Immunohistochemistry (IHC)

A PAAD tissue microarray containing 71 tumor and adjacent non-tumorous pancreatic tissues (Shanghai Xinchao Biotechnology, Shanghai, China; Cat no. HPanA120Su02) was subjected to IHC analysis. IHC staining was performed using a non-biotin detection system (ZSGB-Bio, Beijing, China; Cat no. PV-6000) according to the manufacturer’s instructions. Briefly, tissue sections were deparaffinized in xylene, rehydrated through graded ethanol, and subjected to antigen retrieval in citrate buffer (pH 6.0) at 95 °C for 15 min. Endogenous peroxidase activity was quenched using a peroxidase blocking solution for 10 min at room temperature. Sections were then incubated overnight at 4 °C with a rabbit polyclonal anti-ADGRG6 antibody (1:500; Proteintech, Rosemont, IL, USA, Cat. no. 17774-1-AP). Following PBS washes, sections were incubated with horseradish peroxidase (HRP)-conjugated goat anti-rabbit IgG polymer for 20 min at room temperature. The signal was visualized using a freshly prepared 3,3′-diaminobenzidine (DAB) substrate for 5 min, followed by hematoxylin counterstaining, dehydration, clearing, and mounting. *ADGRG6* expression was semi-quantitatively evaluated based on both staining intensity and the percentage of positive tumor cells, following established scoring criteria: Staining intensity: 0 (no staining), 1 (light yellow), 2 (brown-yellow), 3 (dark brown). Positive cell proportion: 0 (<5%), 1 (5~25%), 2 (26~50%), 3 (51~75%), 4 (>75%). A composite score was calculated by multiplying intensity and proportion scores (range: 0–12). The median composite score (6) was used as the cutoff point to define high expression (>6) and low expression (≤6) groups. To minimize subjectivity, two experienced pathologists independently assessed all stained sections in a double-blinded manner, without access to any clinical or outcome information. In cases where their scores differed by ≥2 points, a consensus score was determined after joint re-evaluation and discussion using corresponding H&E-stained sections. Clinicopathological characteristics of the TMA cohort (including age, sex, tumor size, grade, TNM stage, and AJCC stage) are provided in Supplementary Table S1.

### 2.2. Cell Culture and Transfection

AsPC-1 and BxPC-3 cell lines were purchased from the Chinese Academy of Sciences Cell Bank and cultured in DMEM + 10% FBS (Meilunbio, Dalian, China, Cat. no. MA0212 and PWL217). Small interfering RNA (siRNA) targeting *ADGRG6* (si-ADGRG6, sense strand: 5′-ccaagcaauaaugaaucguautt-3′; antisense strand: 5′-auacgauucauuugcuuggtt-3′) and a negative control (si-NC, sense strand: 5′-uucuccgaacgugucacgutt-3′; antisense strand: 5′-acgugacacguucggagaatt-3′) were synthesized (Sangon Biotech, Shanghai, China) and transfected using Lipofectamine^TM^; 3000 (Invitrogen, Carlsbad, CA, USA, Cat. no. L3000015) per manufacturer’s protocol.

### 2.3. 3D Spheroid Cultivation Protocol

Transfected AsPC-1 and BxPC-3 cells underwent harvesting and resuspension in complete culture medium to achieve a 5 × 10^3^ cells/mL concentration. Using pipettes operated at reduced dispensing velocity (<50 µL/s), 40 µL aliquots of cell suspension were carefully introduced into individual wells of GravityPLUS^TM^; Hanging-Drop Plates via SureDrop^TM^; inlet funnels (InSphero, Schlieren, Switzerland, Cat. no. ISP-06-010). Bottom plate reservoirs received humidifier pads soaked in sterile 0.5 × PBS to minimize evaporation. Incubation proceeded at 37 °C within 5% CO_2_ environment, maintaining >95% relative humidity. After spheroid establishment (2–4 days), formed microtissues underwent transfer to pre-moistened GravityTRAP^TM^; Tissue Receiver Plates through addition of 70 µL complete medium per well at a controlled dispensing velocity ≤ 10 µL/s. Culture medium underwent replacement at 48-h intervals to sustain appropriate nutritional environments. Spheroid documentation occurred at 3, 7, 14, and 21-day timepoints through inverted microscopy. Diameter measurements were performed using ImageJ analysis software (NIH, Bethesda, MD, USA, version 2.14.0), ensuring a minimum of 6 replicate spheroids per treatment group underwent quantification.

### 2.4. Cell Proliferation Assay

Transfected cells were seeded in 96-well plates and cultured for 1~4 days. Cell viability was assessed via CCK-8 (Meilunbio, Dalian, China, Cat. no. MA0218) with absorbance measured at 450 nm using a BioTek Synergy2 microplate reader (Winooski, VT, USA).

### 2.5. Wound-Healing Assay

Cells were grown to 90% confluence, scratched with a pipette tip, washed, and cultured in fresh DMEM. Images were taken at 0 and 24 h to assess wound closure.

### 2.6. Transwell Migration Assay

Migration was evaluated using Transwell chambers (Corning, NY, USA). Cells (1 × 10^5^) were seeded in serum-free medium in the upper chamber; 10% FBS DMEM was added to the lower chamber. After 24 h, migrated cells were fixed, stained (0.5% crystal violet), imaged, and counted.

### 2.7. RT-qPCR

Total RNA was extracted using TRIzol (Invitrogen, Carlsbad, CA, USA, Cat. no. 15596026CN) and reverse-transcribed using an M-MLV Kit (Promega, Madison, WI, USA, Cat. no. D1301). qPCR was conducted with SYBR Green (Takara, Tokyo, Japan, Cat. no. RR420L) on an ABI 7500 system. Primer sequences: *ADGRG6*, F: 5′-TGTCGTTAATATCAGTTTTCACC-3′, R: 5′-TATGTAGCCTCAAGCCTTCA-3′; *STAT6*, F: 5′-CTTTCCGGAGCCACTACAAG-3′, R: 5′-AGGAAGTGGTTGGTCCCTTT-3′; *GATA3*, F: 5′-GAACCGGCCCCTCATTAAG-3′, R: 5′-ATTTTTCGGTTTCTGGTCTGGAT-3′; *β-actin*, F: 5′-ACCGAGCGCGGCTACAG-3′, R: 5′-CTTAATGTCACGCACGATTTCC-3′. PCR conditions: 95 °C for 3 min, then 40 cycles of 95 °C for 10 s, 60 °C for 31 s. Relative expression was calculated using the 2^−ΔΔCt^ method.

### 2.8. Western Blot

Total protein lysates were prepared from AsPC-1 cells transfected with si-ADGRG6 or si-NC for 48 h. Blots were probed using anti-ADGRG6 (1:1000, Proteintech, Rosemont, IL, USA), anti-phospho-NF-κB p65 (Ser468) (1:1000, Proteintech), anti-NF-κB p65 (1:1000, Proteintech), anti-STAT6 (1:1000, Proteintech), anti-phospho-STAT6 (Tyr641) (1:1000, Proteintech), anti-GATA3 (1:1000, Proteintech), and anti-GAPDH (1:3000, Proteintech) antibodies. Bands were visualized using Enhanced Chemiluminescence (ECL, Meilunbio, Dalian, China) and imaged with a ChemiDoc^TM^; Imaging System (Bio-Rad, Hercules, CA, USA). Densitometric quantification was performed using ImageJ, with ADGRG6 intensity normalized to GAPDH.

### 2.9. ELISA

To quantify cytokine secretion, supernatants from AsPC-1 and BxPC-3 cells were collected 48 h after siRNA transfection, centrifuged at 1500× *g* for 10 min, and stored at −80 °C. The concentrations of IL-6 and IL-8 were measured using commercial human ELISA kits (Human IL-6 Quantikine ELISA Kit, Cat. no. MM-0049H2, Meimian, Yancheng, China; Human IL-8 Quantikine ELISA Kit, Cat. no. MM-1558H2, Meimian, Yancheng, China) according to the manufacturer’s instructions. Briefly, 100 µL of standards or samples were added to each well of a pre-coated 96-well plate and incubated for 2 h at room temperature. After washing, detection antibody and streptavidin-HRP were added sequentially, and color development was achieved using TMB substrate. Absorbance was measured at 450 nm using a microplate reader (BioTek, Winooski, VT, USA). Cytokine concentrations were calculated from standard curves and normalized to total protein concentration. All assays were performed in triplicate.

### 2.10. Zebrafish Xenograft Model

At 48 h post-fertilization (hpf), zebrafish (*Danio rerio*) larvae were anesthetized with 0.16 mg/mL tricaine (Sigma-aldrich, MO, USA, Cat. no. E10521) and injected into the yolk sac with 150~200 CM-Dil (Invitrogen, OR, USA, Cat. no. C7000) pre-labeled AsPC-1 cells transfected with either si-ADGRG6 or si-NC per larva. Post-injection, larvae were incubated at 32 °C for 48 h. For each group, at least 30 larvae were injected to ensure a minimum of 10 successful engraftments. More than 10 larvae per group were randomly selected and imaged using a stereomicroscope, Leica. Tumor fluorescence area was quantified using ImageJ software (NIH, Bethesda, MD, USA, version 2.14.0) for further analysis. Euthanasia followed the US Food and Drug Administration (FDA) and the AVMA *Guidelines on Euthanasia* via ice water immersion on 5 dpf [24]. Cardiac arrest was confirmed in a stereomicroscope Leica (Leica, Wetzlar, Germany, M205C) before bleaching in 10% sodium hypochlorite (Aladdin, Shanghai, China, Cat. no. S291945) [25].

### 2.11. Animal Experiments

Female athymic BALB/c nude mice aged four to five weeks (body weight: 18–22 g) were procured from Xiamen Fudexin Biotechnology Co., Ltd. (Xiamen, China). The Laboratory Animal Welfare and Ethics Committee of Fujian Provincial Hospital granted approval for all experimental procedures (IACUC-FRH-SL-20250207 [0561]). Animals were maintained within specific pathogen-free (SPF) housing under regulated environmental parameters (temperature: 20–30 °C; humidity: 60–80%) with unrestricted access to SPF rodent diet and sterilized water. Transfected AsPC-1 cells (si-*ADGRG6*, si-Cont control siRNA, or NC negative control) underwent subcutaneous injection into the right flank region of individual mice at 5 × 10^6^ cells per injection site (*n* = 6 animals per experimental group). Tumor development was assessed at three-day intervals through caliper measurements of minimum diameter (A) and maximum diameter (B). Volume calculations (V) employed the formula V = (A^2^ × B)/2. Body weight documentation occurred concurrently with tumor measurements. Following a 30-day experimental period, animals received isoflurane anesthesia (2% via inhalation route) before euthanasia through cervical dislocation, with confirmation via cessation of cardiovascular and respiratory functions. Tumor tissues underwent immediate excision and weight determination post-euthanasia.

### 2.12. Bioinformatics Databases

For expression analysis, GEPIA (http://gepia.cancer-pku.cn/index.html, accessed on 12 November 2025) was used to evaluate *ADGRG6* mRNA and protein expression levels and survival in PAAD patients, integrating The Cancer Genome Atlas (TCGA) and Genotype-Tissue Expression (GTEx) data. UALCAN (http://ualcan.path.uab.edu/, accessed on 12 November 2025) is a web-based platform for analyzing cancer OMICS data derived from TCGA, MET500, CPTAC, and CBTTC sources [26]. It was used to analyze expression in relation to clinicopathological subgroups in this study. “Normal” tissues refer to non-malignant adjacent tissues from the TCGA-CPTAC cohort and are not annotated for patient sex or comorbidity status. TIMER (https://cistrome.shinyapps.io/timer/, accessed on 12 November 2025) is a comprehensive database used for systematic analyses of immune infiltration in various tumors [27]. The Kaplan–Meier plotter (https://kmplot.com/analysis/, accessed on 10 May 2024) was used to explore the prognostic value of ADGRG6 based on immune cell status. TISCH2 (http://tisch.comp-genomics.org/, accessed on 21 May 2024) is a single-cell RNA-seq database focused on TME landscapes [28]. From the eight pancreatic cancer datasets available in TISCH, we selected PAAD_CRA001160 and PAAD_GSE154778 for downstream TME analysis due to their comprehensive clinical and molecular profiles and better annotation [29,30]. GeneMANIA (http://www.genemania.org, accessed on 12 November 2025) was used to predict gene function based on co-expression and interaction networks. LinkedOmics (http://www.linkedomics.org/login.php, accessed on 12 November 2025) provided additional transcriptomic correlation analysis using TCGA data from 32 cancers and 10 CPTAC cohorts [31]. IHC images of ADGRG6 in PAAD and normal pancreas tissues were randomly obtained from the Human Protein Atlas (HPA) database (https://www.proteinatlas.org/, accessed on 12 November 2025). The KEGG pathway enrichment of *ADGRG6* co-expressed genes was conducted using the LinkInterpreter module of the Linkedomics database. Pearson correlation analysis between *ADGRG6* and representative signaling genes (*NFKB1*, *RELA*, *STAT6*, and *GATA3*) was performed using expression data from 178 TCGA-PAAD clinical samples. Correlation coefficients (R values) and *p* values were calculated and visualized using GraphPad Prism 8.

### 2.13. Statistical Analysis

Data was analyzed using SPSS (version 22.0, IBM, Armonk, NY, USA) and web-based tools. Survival analysis was performed with Kaplan–Meier plots (https://kmplot.com/analysis/, accessed on 10 May 2024). Association with immune markers were tested via Spearman’s correlation (TIMER2.0 database, http://timer.cistrome.org/, accessed on 12 November 2025). Univariate and multivariate Cox regression assessed prognostic factors. Student’s *t*-test or ANOVA with Bonferroni’s post hoc tests were used. Experiments were repeated ≥3 times. *p* < 0.05 was considered statistically significant.

## 3. Results

### 3.1. ADGRG6 Is Consistently Upregulated in PAAD Across Clinical and Molecular Subgroups

**Figure 1:**
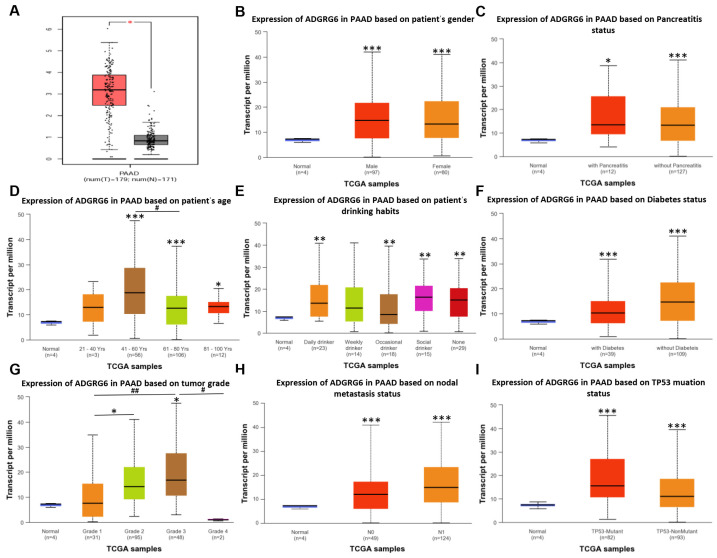
Expression levels of *ADGRG6* mRNA in PAAD and clinical subgroups (GEPIA and UALCAN). (**A**) Comparison of *ADGRG6* mRNA expression between PAAD tissues (*n* = 179) and normal tissues (*n* = 171) from the GEPIA database (TCGA + GTEx). (**B**–**I**) UALCAN-based subgroup analysis of *ADGRG6* expression levels in PAAD samples stratified by sex (**B**), pancreatitis status (**C**), age (**D**), drinking habits (**E**), diabetes status (**F**), tumor grade (**G**), lymph node metastasis (**H**), and *TP53* mutation status (**I**). The “normal” group in UALCAN (*n* = 4) includes adjacent non-tumor tissues, without subgroup annotations. The comparison to the “normal” group in the UALCAN analysis should be interpreted with caution due to the small size of the normal cohort (*n* = 4). “Drinking status” data is incomplete (missing in 80 samples), and comparisons among subgroups should be interpreted cautiously. Subtype descriptions of “Tumor Grade”: Grade 1-Well differentiated (low grade), Grade 2-Moderately differentiated (intermediate grade), Grade 3-Poorly differentiated (high grade), Grade 4-Undifferentiated (high grade). Pathologic descriptions of “Nodal Metastasis Status”: N0-No regional lymph node metastasis, N1-Metastases in 1 to 3 axillary lymph nodes. Data are presented as Mean ± SD. Statistical analysis was performed using Student’s *t*-test for two-group comparisons and one-way ANOVA followed by Bonferroni’s post hoc test. “*” indicates comparison with the control group; “#” indicates significance between experimental groups (* *p* < 0.05, ** *p* < 0.01, *** *p* < 0.001; ^#^ *p* < 0.05, ^##^ *p* < 0.01).

To investigate the expression pattern of *ADGRG6* in PAAD, we first examined its mRNA levels in tumor and normal pancreatic tissues using the GEPIA database, which integrates RNA-seq data from TCGA and GTEx. The results showed that *ADGRG6* was significantly upregulated in PAAD tissues (Tumor, T, *n* = 179) compared with the normal population (Normal, N, *n* = 171) (Figure 1A, * *p* < 0.05). To further evaluate whether this elevated expression was consistent across various clinical contexts, we analyzed *ADGRG6* mRNA levels in several patient subgroups using the UALCAN database, which utilizes TCGA-CPTAC data. Subgroups included sex, pancreatitis status, age, drinking habits, diabetes status, tumor grade, lymph node metastasis, and *TP53* mutation status (Figure 1B–I). It is important to note that the normal cohort in this UALCAN-derived dataset is limited (*n* = 4), which warrants caution in interpreting the magnitude of this difference. To assess the validity of differential expression calls within this specific dataset, we performed an internal control analysis using well-established PAAD driver genes within the same platform. As shown in Supplementary Figure S1, the expression patterns (*KRAS* overexpression; *TP53* and *SMAD4* downregulation) align with the canonical molecular landscape of PAAD [32,33]. This internal control supports the utility of the dataset for identifying biologically relevant trends, thereby lending credence to the consistent overexpression of *ADGRG6* observed across PAAD subgroups.

In general, *ADGRG6* mRNA expression was significantly elevated in all clinical subgroups compared to normal tissues (all *p* < 0.05), with the exception of the two “21~40 Yrs” and “weekly drinker” groups (*p* = 0.44 and *p* = 0.068, respectively). However, pairwise comparisons among PAAD subgroups themselves (e.g., male vs. female, diabetic vs. non-diabetic, age groups) did not yield statistically significant differences (*p* > 0.05 in all cases). A full list of comparisons and *p*-values is provided in Supplementary Table S3.

For the drinking status variable, we further found that metadata was unavailable for 80 of the TCGA-PAAD samples, which reduces the reliability of this clinical subgroup. Although the “non-drinker” group exhibited the second-highest mean *ADGRG6* expression, comparisons among drinking groups were not statistically significant, suggesting that drinking habits are unlikely to be a biologically meaningful determinant of *ADGRG6* expression in PAAD.

Despite extensive subgroup analyses, *ADGRG6* expression showed consistent elevation across clinical and molecular groups, with minimal inter-subgroup variation. This supports its potential utility as a broadly applicable diagnostic or prognostic biomarker in PAAD.

**Figure 2:**
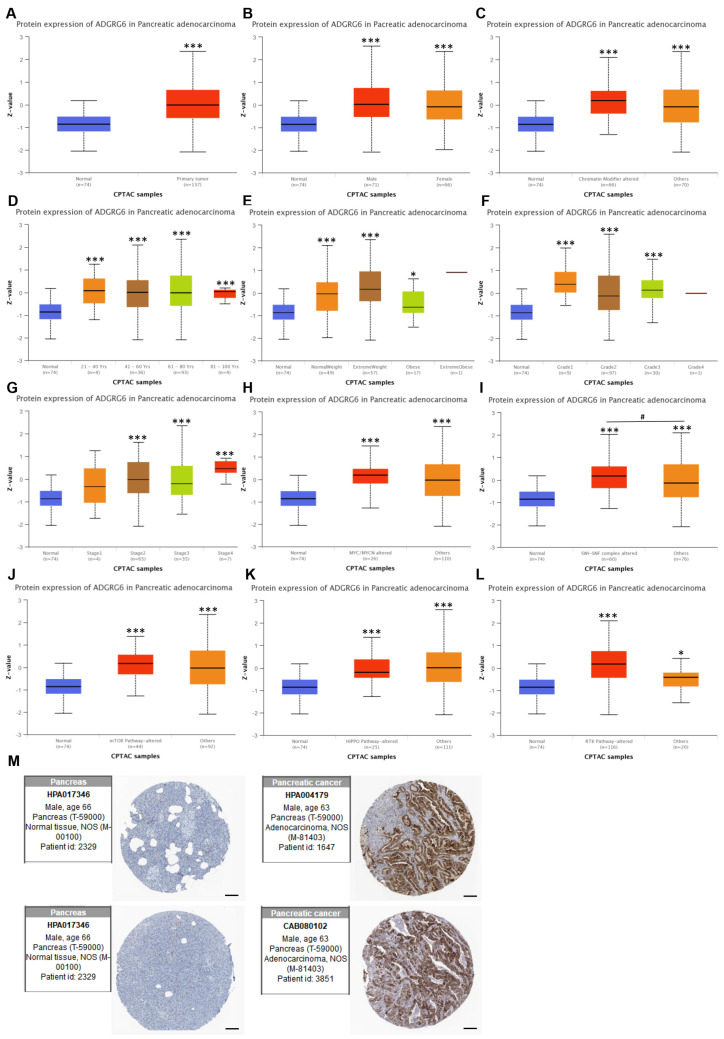
Protein expression of ADGRG6 in PAAD based on UALCAN and HPA databases. (**A**) Comparison of ADGRG6 protein expression between PAAD tissues (*n* = 137) and normal pancreatic tissues (*n* = 74) using the UALCAN database (CPTAC dataset). (**B**–**L**) Subgroup analyses of ADGRG6 protein expression stratified by sex (**B**), chromatin modifier alteration status (**C**), age (**D**), weight (**E**), tumor grade (**F**), tumor stage (**G**), *MYC/MYCN* alteration (**H**), SWI/SNF complex alteration (**I**), and activity status of the mTOR (**J**), Hippo (**K**), and RTK (**L**) signal pathways. Each subgroup was compared to normal tissues. The “others” group in (**C**,**H**–**L**) refers to patients without the specific mutation or alteration listed. (**M**) Representative IHC staining images from the HPA database showing ADGRG6 expression in normal pancreatic tissue and PAAD tissue. Scale Bar: 200 µm. Data are presented as Mean ± SD. Statistical analysis was performed using Student’s *t*-test for two-group comparisons and one-way ANOVA followed by Bonferroni’s post hoc test. “*” indicates comparison with the control group; “#” indicates significance between experimental groups (* *p* < 0.05, *** *p* < 0.001; ^#^ *p* < 0.05).

We next analyzed ADGRG6 protein-level expression using the UALCAN proteomics module (based on CPTAC). Consistent with transcriptomic results, ADGRG6 protein levels were significantly elevated in PAAD tissues compared to normal pancreatic tissues (Figure 2A). Subgroup analyses were further conducted according to clinical and molecular parameters, including sex, chromatin modifier alterations, age, weight, tumor grade, *MYC/MYCN* alterations, SWI/SNF complex alterations, and activity status of mTOR, Hippo, and receptor tyrosine kinase (RTK) signaling pathways (Figure 2B–L), although inter-subgroup differences were not always statistically significant. In subgroup panels such as chromatin modifiers, *MYC/MYCN* alterations, the “others” group refers to patients without these mutations or with unannotated genetic status in the CPTAC dataset.

Additionally, IHC data from the HPA database corroborated these findings, showing increased ADGRG6 staining intensity in PAAD tissues compared to normal pancreatic tissues (Figure 2M). Taken together, these findings demonstrate that ADGRG6 is broadly and consistently upregulated in PAAD tissues at both the mRNA and protein levels, across a variety of clinical and molecular contexts. However, its expression does not appear to be driven by specific clinicopathological features, suggesting it may serve as a general molecular hallmark of PAAD rather than a subgroup-specific marker. These results prompted further evaluation of the clinical significance and biological role of *ADGRG6* in PAAD.

### 3.2. High ADGRG6 Expression Predicts Poor Overall Survival in Patients with PAAD

**Figure 3:**
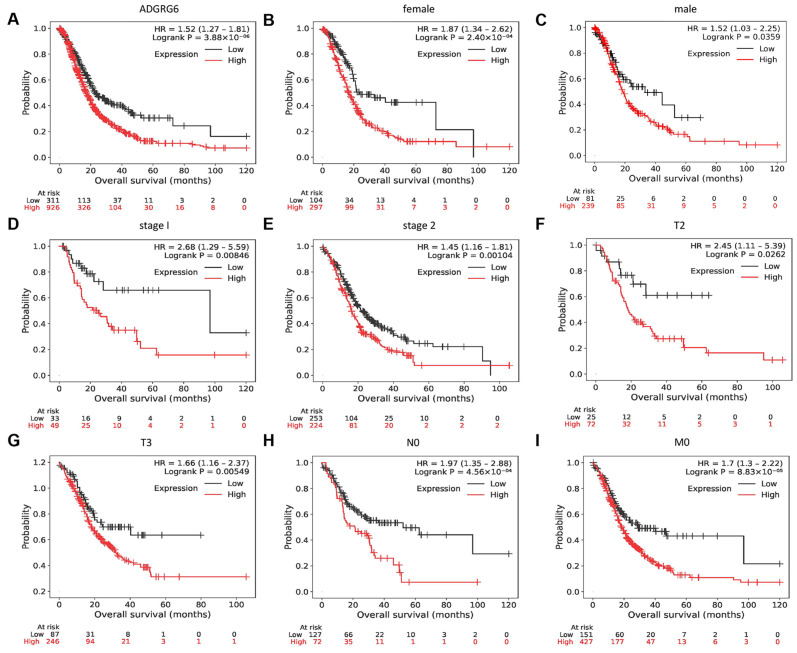
Prognostic value of *ADGRG6* expression in PAAD. (**A**) Kaplan–Meier survival curves showing OS relative to *ADGRG6* expression. (**B**–**I**) Subgroup OS analyses, including (**B**,**C**) gender, (**D**,**E**) stage 1–2, (**F**) T2 (tumor size > 2 cm but ≤4 cm), (**G**) T3 (tumor size > 4 cm), (**H**) N0 (no regional lymph node metastasis), and (**I**) M0 (no distant metastasis).

Given its robust overexpression in PAAD tissues, we next evaluated whether ADGRG6 expression levels were associated with patient outcomes. Kaplan–Meier survival curve analysis was used to evaluate the correlation between *ADGRG6* expression and the overall survival (OS) of patients with PAAD. High *ADGRG6* expression was associated with a worse OS rate for patients with PAAD (Figure 3A). Subsequently, the association between *ADGRG6* expression and OS in different subgroups was investigated. Patients with high *ADGRG6* expression had a worse OS in multiple subgroups, including sex, cancer stages 1–2, T2–3 (tumor size >2 cm but ≤4 cm, >4 cm), N0 (no regional lymph node metastasis), and M0 (no distant metastasis) (Figure 3B–I). Collectively, these findings suggested that *ADGRG6* can be used as a biomarker map to predict the prognosis of patients with PAAD.

### 3.3. ADGRG6 Expression Is Enriched in Malignant and Stromal Cell Populations at the Single-Cell Level of the TME

**Figure 4:**
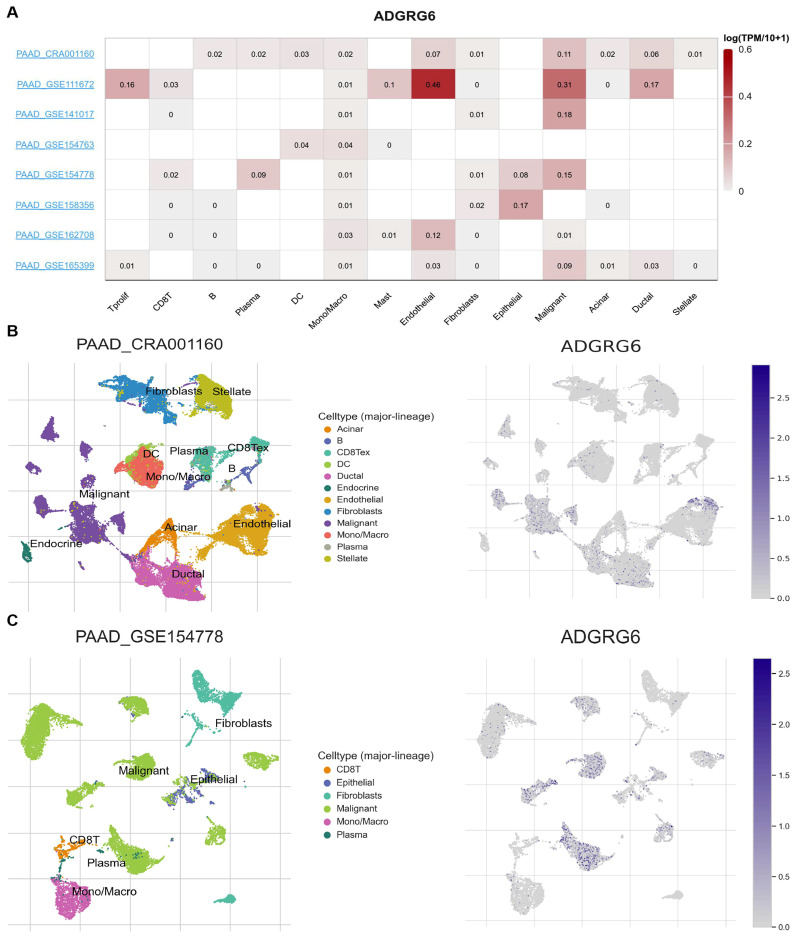
Single-cell analysis of *ADGRG6* in the TME. (**A**) TISCH database analysis of *ADGRG6* expression across different cell types in the TME. (**B**,**C**) The distribution of *ADGRG6* expression in various immune and stromal cell types in the PAAD_CRA001160 and PAAD_GSE154778 datasets. The left panels (**B**,**C**) depict the Uniform manifold approximation and projection (UMAP) of single-cell transcriptome data with cell typing (major lineages) in the two datasets. The right panels (**B**,**C**) show the expression of the *ADGRG6* gene in different cell types (major lineages) within the two datasets.

To further investigate the cell-type-specific distribution of *ADGRG6* in the TME of PAAD, we analyzed single-cell RNA sequencing (scRNA-seq) datasets using the TISCH database. The expression of *ADGRG6* across various immune and stromal cell types in different datasets is illustrated in Figure 4A. Among the eight available PAAD datasets, we specifically focused on PAAD_CRA001160 and PAAD_GSE154778, as these two datasets provide comprehensive clinical and molecular profiles, offering valuable insights into the complexity of the TME, including tumor, immune, and stromal cell populations. Both datasets utilized a high-quality scRNA-seq platform (10x Genomics), enabling a detailed examination of *ADGRG6* expression at the single-cell level.

The PAAD_CRA001160 dataset provides scRNA-seq data from 24 PAAD tumor samples and 11 control pancreases with a total of 41,986 cells from PAAD tumors and 15,544 cells from control tissues. The PAAD_GSE154778 dataset includes 16 patients, with 10 primary tumor tissues and 6 metastatic biopsies (from liver or omentum). A total of 8000 cells were obtained from the 10 primary tumors and 6926 cells from the 6 metastasis samples. To visualize the distribution of *ADGRG6* expression in different immune and stromal cell types, we employed Uniform Manifold Approximation and Projection (UMAP) plots (Figure 4B,C). In the PAAD_CRA001160 dataset, *ADGRG6* was primarily expressed in malignant cells, endothelial cells, ductal cells, and dendritic cells (DCs) (Figure 4B). Similarly, in the PAAD_GSE154778 dataset, *ADGRG6* was expressed in malignant cells, plasma cells, epithelial cells, and CD8+ T cells (Figure 4C). These findings suggested that *ADGRG6* is not only present in malignant cells but also in various immune and stromal cells within the TME, contributing to the heterogeneity observed in PAAD.

### 3.4. ADGRG6 Expression Correlates with Immune Cell Infiltration and Immune-Related Signatures

To investigate the functional significance of ADGRG6 in the PAAD immune microenvironment, we analyzed the correlation between *ADGRG6* expression and molecular signatures of various immune cell subsets using the TIMER and GEPIA database platforms. Spearman’s rank correlation analysis was performed to determine the correlation coefficient (Rho) and statistical significance (*p*-value).

The results showed that *ADGRG6* was significantly positively correlated with the mast cell marker genes *CPA3* and *KIT*, with a relatively strong correlation with *KIT* (Rho = 0.285, *p* < 0.001 in the TIMER database; Rho = 0.28, *p* < 0.001 in the GEPIA database). In M1 macrophages, there was also a significant positive correlation between the expression of *CD86* and *CD80* genes and *ADGRG6* (Rho values were between 0.15 and 0.25, *p* < 0.05 for both). For the type I interferon response subset of neutrophils, the marker genes *IFIT1* and *RSAD2* were significantly correlated with *ADGRG6* (Rho was approximately 0.2 to 0.27, *p* < 0.01 to *p* < 0.001). In addition, the key transcription factors *GATA3*, *STAT6*, *STAT3*, and *BATF* of the helper T cell subsets Th2 and Th17 were highly positively correlated with *ADGRG6*, especially *STAT6*, which had the strongest correlation with *ADGRG6* (Rho > 0.5, *p* < 0.001) (Table 1).

In summary, the expression level of *ADGRG6* was significantly positively correlated with the specific gene markers of multiple immune cell types, suggesting that *ADGRG6* may play an important role in the regulation of immune cells and the immune microenvironment. Given these associations with immune-related markers, we next explored whether *ADGRG6* directly contributes to tumor progression and immune signaling.

### 3.5. Clinical Validation of ADGRG6 Expression in a PAAD Tissue Microarray Cohort

To validate the clinical significance of ADGRG6 expression, we performed IHC staining on a PAAD TMA containing 71 tumor samples. Elevated ADGRG6 expression was observed in 49 cases (69.01%), while reduced expression was detected in 22 cases (30.99%). Correlation analysis of clinicopathological parameters demonstrated that high ADGRG6 expression was significantly associated with multiple unfavorable tumor parameters, including larger tumor size (*p* < 0.05), higher pathological grade (*p* < 0.001), presence of organ invasion (*p* < 0.05), advanced TNM stage (*p* < 0.01), and higher AJCC stage (*p* < 0.05). Conversely, no significant correlations were identified between ADGRG6 expression and patient gender, age, or nerve invasion (Table 2).

**Figure 5:**
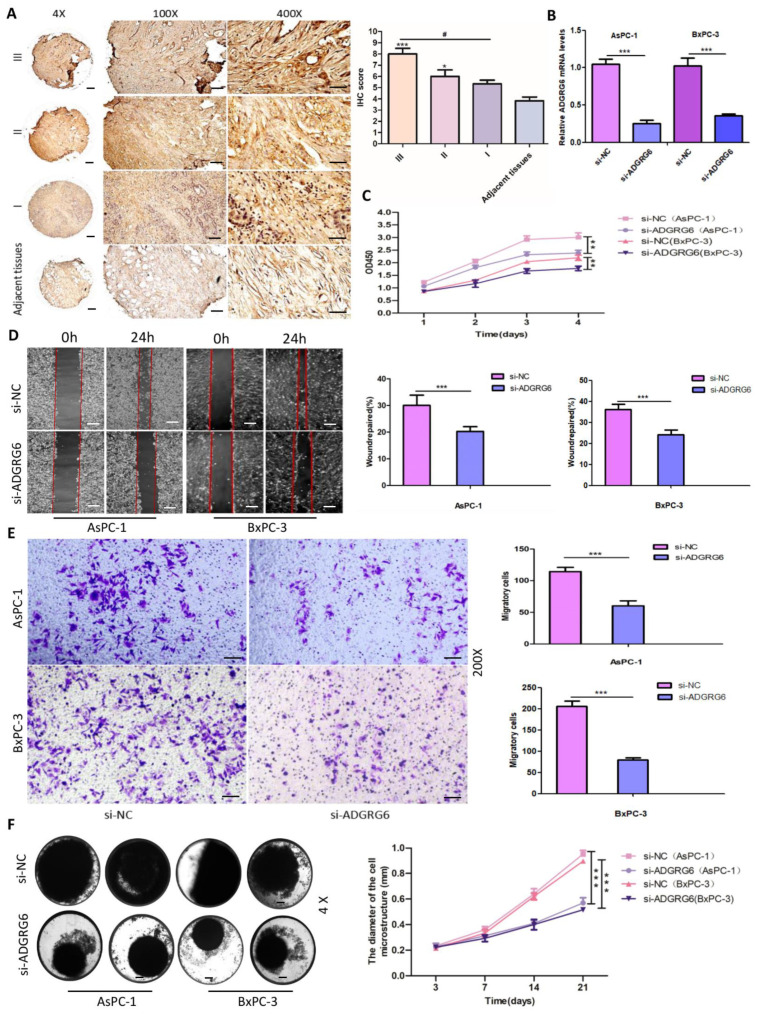
*ADGRG6* silencing suppresses PAAD cell proliferation, migration, and invasion in vitro. (**A**) IHC staining of *ADGRG6* in PAAD tissues based on tissue microarray analysis, showing higher expression in advanced TNM stages. Scale Bar: 200 µm. (**B**) *ADGRG6* mRNA levels in AsPC-1 and BxPC-3 post si-*ADGRG6*. (**C**) Cell proliferation of si-*ADGRG6*-transfected AsPC-1 and BxPC-3 measured by CCK-8 assay. (**D**) Wound-healing assay demonstrating reduced migration capacity in si-*ADGRG6*-transfected AsPC-1 and BxPC-3 cells. Scale Bar: 50 µm. (**E**) Transwell invasion assays (200×) confirming decreased invasive ability post-knockdown. Scale Bar: 50 µm. (**F**) 3D spheroid culture assays demonstrating impaired spheroid growth in si-*ADGRG6* cells, quantified by spheroid diameters across 21 days (4×). Scale Bar: 200 µm. Data are presented as Mean ± SD. Statistical analysis was performed using Student’s *t*-test for two-group comparisons and one-way ANOVA followed by Bonferroni’s post hoc test. “*” indicates comparison with the control group; “#” indicates significance between experimental groups (** *p* < 0.01, *** *p* < 0.001; ^#^ *p* < 0.05).

To further account for potential confounding among these variables, a multivariate logistic regression analysis was conducted, with ADGRG6 expression status (high vs. low) as the dependent variable, and gender, age, pathological grade, tumor size, organ invasion, nerve invasion, TNM stage, and AJCC stage as independent variables. indicated that pathological grade (OR = 10.026, 95% CI: 1.793–56.047, *p* = 0.009) and tumor size (OR = 20.457, 95% CI: 2.133–196.237, *p* = 0.009) were independent risk factors associated with high ADGRG6 expression. In contrast, gender, age, organ invasion, nerve invasion, TNM stage, and AJCC stage showed no statistically significant association (all *p* > 0.05). TMA-based IHC scoring also revealed that ADGRG6 expression positively correlated with TNM staging, with maximal staining intensity observed in stage III specimens (Figure 5A). Taken together, these findings suggest that increased ADGRG6 expression is closely associated with higher pathological grade and larger tumor burden, both key indicators of aggressive tumor behavior in PAAD. This supports a potential role for ADGRG6 as a biomarker of malignant progression and provides a rationale for subsequent mechanistic and functional validation.

### 3.6. ADGRG6 Promotes PAAD Cell Proliferation, Migration, and Invasion In Vitro and In Vivo

To examine *ADGRG6*’s functional role in PAAD cellular proliferation, migration, and invasion processes, we conducted targeted silencing of *ADGRG6* mRNA in AsPC-1 and BxPC-3 pancreatic cancer cell lines through specific siRNAs (si-*ADGRG6*), utilizing si-NC as negative control. Quantitative verification established effective *ADGRG6* silencing at mRNA levels across both cell lines (Figure 5B). CCK-8 assays showed that *ADGRG6* silencing markedly inhibited growth in AsPC-1 and BxPC-3 cells throughout a 4-day timeframe (Figure 5C, ** *p* < 0.01). Wound-healing experiments indicated that si-*ADGRG6* treatment produced substantially decreased wound closure rates versus si-NC controls across both cell lines within 24 h (Figure 5D, *** *p* < 0.001). Transwell invasion studies additionally revealed that *ADGRG6* silencing generated substantial decreases in invasive potential for both AsPC-1 and BxPC-3 cells (Figure 5E, *** *p* < 0.001). To mimic the physiological 3D microenvironment, we further conducted spheroid formation assays. si-*ADGRG6*-transfected cells formed smaller and less compact spheroids than control cells, and quantitative analysis over 21 days verified a significant decrease in spheroid diameter (Figure 5F, *** *p* < 0.001), suggesting impaired 3D proliferative capacity.

**Figure 6:**
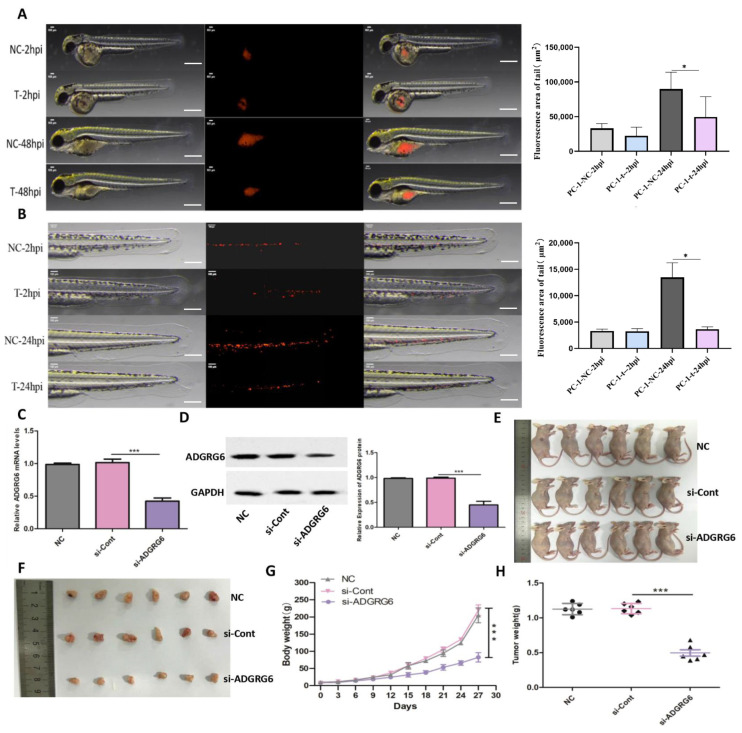
In vivo evidence of *ADGRG6* oncogenic function in zebrafish and murine xenograft models. (**A**) Representative fluorescence microscopy images of zebrafish xenografts injected with CM-DiI-labeled AsPC-1 cells (si-NC vs. si-*ADGRG6*) at 48 h post-injection (hpi), showing reduced tumor fluorescence area in si-*ADGRG6* xenografts. Scale Bar: 300 µm. (**B**) Migration distance of tumor cells in zebrafish xenografts at 24 hpi, significantly reduced upon *ADGRG6* knockdown. Fluorescence (red) resulting from CM-Dil labeling was used to monitor the behavior of the cells in the zebrafish model. (**C**) Relative *ADGRG6* mRNA and (**D**) protein levels in AsPC-1 cells transfected with siRNA. (**E**) Representative images of mice in vivo tumorigenesis assay. (**F**) Representative images of excised tumors from the tumor-bearing mice. (**G**) Body weight of mice across groups was measured every three days for each mouse and the growth curve was plotted (*n* = 6). (**H**) Comparison of the tumor weight (*n* = 6). Data are presented as Mean ± SD. Statistical analysis was performed using Student’s *t*-test for two-group comparisons and one-way ANOVA followed by Bonferroni’s post hoc test. * *p* < 0.05, *** *p* < 0.001.

For in vivo validation of these laboratory findings, we implemented a zebrafish xenograft system. CM-DiI-labeled AsPC-1 cells treated with si-*ADGRG6* or si-NC underwent injection into zebrafish, with fluorescence microscopy tracking cellular proliferation and migration. At 48 h post-injection (hpi), fluorescence areas representing cellular proliferation showed significant decreases in si-*ADGRG6* groups versus si-NC controls (Figure 6A, * *p* < 0.05). Migration studies revealed that si-*ADGRG6*-treated cells displayed reduced motility within zebrafish at 24 hpi (Figure 6B, * *p* < 0.05). For murine xenograft experiments, si-*ADGRG6*-transfected AsPC-1 cells were subcutaneously injected into nude mice. Knockdown efficiency was verified at both mRNA and protein levels prior to injection (Figure 6C,D, original Western blot images provided in Supplementary Figure S2). Mice receiving si-*ADGRG6*-treated AsPC-1 cells generated substantially smaller tumors versus NC or si-Cont (control siRNA) cohorts (Figure 6E,F). During the 30-day monitoring period, body weight remained comparable between the NC and si-NC groups. In contrast, mice in the si-*ADGRG6* group exhibited a significant decrease in body weight (Figure 6G), suggesting potential systemic effects associated with sustained *ADGRG6* knockdown. Final tumor weight measurements showed substantial decreases in si-*ADGRG6* cohorts (Figure 6H, *** *p* < 0.001).

Collectively, these results demonstrate that *ADGRG6* exerts potent oncogenic effects in PAAD by promoting cellular proliferation, migration, invasion, and tumor formation, thereby supporting its potential role as a therapeutic target.

### 3.7. ADGRG6 Modulates the NF-κB→STAT6→GATA3 Signaling Axis

**Figure 7:**
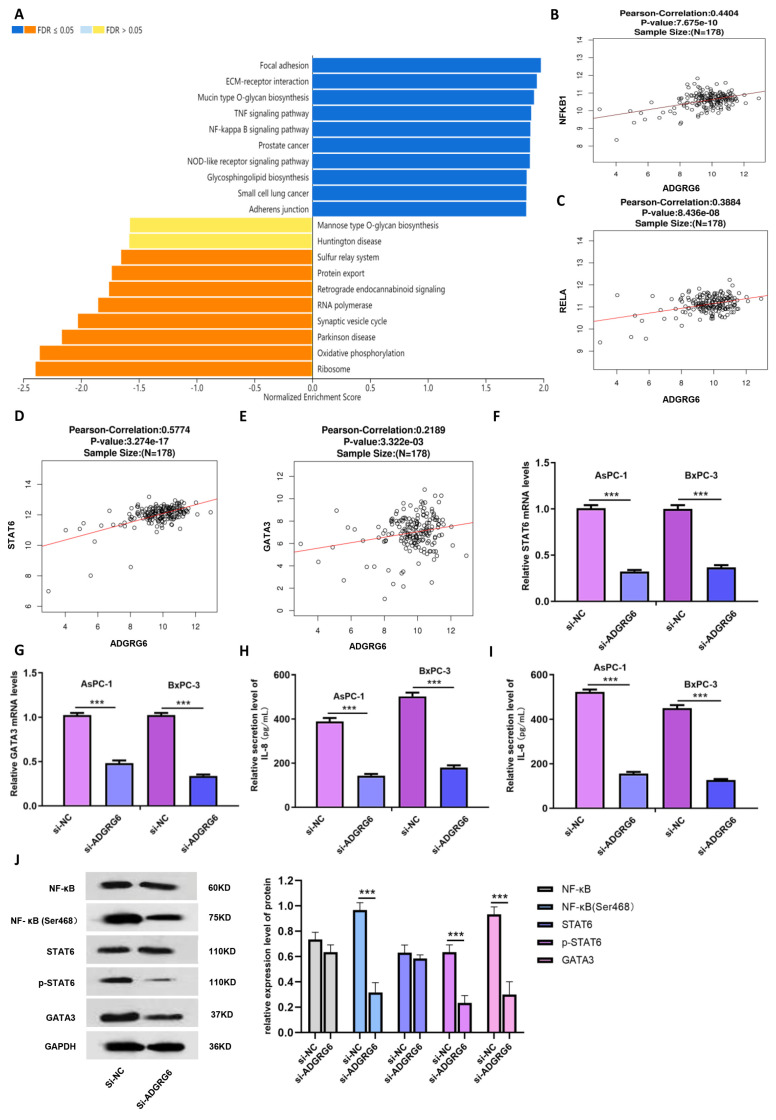
Regulatory Role of *ADGRG6* in the NF-κB→STAT6→GATA3 Signaling Axis. (**A**) KEGG pathway analysis of the gene set co-expressed with *ADGRG6* using the LinkInterpreter module; (**B**–**E**) Pearson correlation analysis between *ADGRG6* and key genes of the signaling axis (*NFKB1*, *RELA*, *STAT6*, and *GATA3*) in 178 clinical samples. Values represent Pearson correlation coefficients and corresponding *p*-values, *n* = 178; (**F**,**G**) Relative expression levels of *STAT6* and *GATA3* genes in the signaling axis in AsPC-1 and BxPC-3 cells after *ADGRG6* knockdown detected by RT-qPCR. Data are presented as mean ± standard deviation; (**H**,**I**) Secretion levels of secretory cytokines IL-6 and IL-8 in AsPC-1 and BxPC-3 cells detected by ELISA; (**J**) Expression and activity of key proteins in the signaling axis after *ADGRG6* knockdown detected by Western blot, *** *p* < 0.001.

To elucidate the molecular mechanisms through which *ADGRG6* promotes PAAD progression, we conducted pathway enrichment analyses using the LinkInterpreter module. KEGG analysis revealed that *ADGRG6* co-expressed genes were significantly enriched in the NF-κB signaling pathway (Figure 7A). Given the previously observed positive correlations between *ADGRG6* and immune-related markers such as STAT6 and GATA3, we hypothesized that *ADGRG6* may influence immune-associated transcriptional regulation via the NF-κB→STAT6→GATA3 axis.

Correlation analyses using clinical transcriptomic data (*n* = 178) confirmed that ADGRG6 expression was significantly and positively correlated with NF-κB pathway core genes (*NFKB1*, *RELA*) as well as with *STAT6* and *GATA3* (Figure 7B–E). To validate these associations experimentally, *ADGRG6* was silenced in AsPC-1 and BxPC-3 cells, and the expression of key pathway genes was quantified by RT-qPCR. Knockdown of *ADGRG6* led to substantial downregulation of *STAT6* and *GATA3* mRNA levels (Figure 7F,G, *** *p* < 0.001). Moreover, ELISA assays revealed that the secretion of downstream proinflammatory cytokines of the NF-κB pathway, IL-6 and IL-8, was markedly reduced in the si-*ADGRG6* group (Figure 7H,I, *** *p* < 0.001).

Western blot analysis further demonstrated that while total NF-κB and STAT6 protein levels remained largely unchanged, their phosphorylated forms (*p*-NF-κB at Ser468 and *p*-STAT6 at Tyr641) were significantly decreased following *ADGRG6* knockdown (Figure 7J). GATA3 protein levels were also reduced, consistent with its transcriptional downregulation, suggesting that *ADGRG6* may regulate GATA3 expression by modulating NF-κB and STAT6 activation.

Together, these findings indicate that *ADGRG6* promotes PAAD progression by activating the NF-κB→STAT6→GATA3 signaling cascade, enhancing the production of inflammatory cytokines such as IL-6 and IL-8, and potentially contributing to tumor–immune crosstalk within the pancreatic tumor microenvironment.

## 4. Discussion

In this study, we tested the hypothesis that *ADGRG6* is overexpressed in PAAD and drives tumor progression through NF-κB/STAT6-mediated signaling and modulation of the TME. Consistent with this premise, our results demonstrated that *ADGRG6* was markedly upregulated in PAAD tissues and cell lines, correlated with aggressive clinicopathological features and poor prognosis, and functionally promoted tumor proliferation, migration, and cytokine secretion across 2D cultures, 3D spheroids, zebrafish xenografts, and murine models. These findings position *ADGRG6* as a potential oncogenic regulator linking intracellular signaling activation with immune modulation in PAAD.

Our findings align with and extend prior evidence implicating *ADGRG6* in tumor progression across multiple cancer types. For instance, *ADGRG6* promotes breast cancer growth upon progesterone stimulation [21], serves as a mutation marker for bladder cancer recurrence and immunotherapy monitoring [34,35,36], and drives colorectal cancer proliferation via HDAC2 and GLI2 signaling [37]. Its role in angiogenesis, which is mediated through the cAMP-PKA-CREB pathway and VEGFR2 regulation [38], further underscores its multifaceted oncogenic potential. Importantly, Wu et al. [23] recently identified *ADGRG6* as a prognostic biomarker that stabilizes mutant p53 and activates the EGFR/NF-κB signaling axis, promoting tumor cell proliferation in a tumor-intrinsic manner. Building upon their findings, our study provides two additional layers of evidence that expand the biological relevance of *ADGRG6*.

First, through clinical validation in a TMA cohort, we confirmed that ADGRG6 protein overexpression correlates with multiple aggressive tumor features, reinforcing its translational potential as a prognostic biomarker. Second, we revealed a previously uncharacterized connection between *ADGRG6* and tumor-immune microenvironmental modulation. Transcriptomic correlation analyses indicated that *ADGRG6* expression is positively associated with immune markers of Th2 and Th17 subsets [39], as well as NF-κB→STAT6→GATA3 signaling components. Single-cell RNA-sequencing data from the TISCH database further showed that *ADGRG6* is expressed not only in malignant epithelial cells but also in endothelial and dendritic cell compartments, suggesting that it may participate in tumor–stroma and tumor–immune crosstalk. Given that endothelial-to-mesenchymal transition (EndMT) [40,41,42,43] and macrophage/Treg-driven immunosuppression [44,45] are central to PAAD progression; the findings raise the possibility that *ADGRG6* contributes to establishing an immunosuppressive niche. Mechanistically, our in vitro data demonstrate that *ADGRG6* knockdown suppresses phosphorylation of NF-κB and STAT6, decreases GATA3 expression, and reduces secretion of IL-6 and IL-8, supporting the existence of an “*ADGRG6*→NF-κB→STAT6→GATA3” regulatory axis that may contribute to Th2-skewed immune polarization [46,47,48].

While these findings highlight an intriguing link between *ADGRG6* signaling and immunoregulatory pathways, several limitations should be acknowledged. Our in vivo validation relied on zebrafish and murine xenograft models, both of which are immunodeficient systems that lack a fully functional adaptive immune response. Therefore, although these models confirm the tumor-suppressive effects of *ADGRG6* silencing on tumor proliferation and invasion, they cannot fully capture the complexity of *ADGRG6*-mediated immune modulation. Consequently, the proposed role of *ADGRG6* in shaping the immunosuppressive PAAD microenvironment should be interpreted as hypothesis-generating rather than conclusive. Future studies employing immunocompetent or humanized mouse models will be essential to delineate the precise mechanisms through which *ADGRG6* influences tumor–immune crosstalk and to evaluate its therapeutic potential in immune-relevant contexts.

Furthermore, the small number of normal pancreatic samples available in the TCGA-UALCAN dataset (*n* = 4) represents an additional constraint that may affect the statistical robustness of differential expression analyses. This limitation is not unique to our study but reflects a broader challenge in pancreatic cancer transcriptomic research, as histologically normal pancreatic tissues are rarely resected and therefore underrepresented in public datasets. Consequently, the apparent magnitude of *ADGRG6* upregulation should be viewed as indicative rather than definitive. To partially address this concern, we performed an internal validation using canonical PAAD driver genes (*KRAS*, *TP53*, and *SMAD4*), which displayed the expected expression patterns within the same dataset. This consistency suggests that, despite the small normal cohort, the UALCAN platform captures biologically meaningful expression trends. Future analyses integrating normal pancreatic tissues from GTEx and our institutional cohort will be required to confirm the robustness of *ADGRG6* dysregulation under more balanced sample conditions.

Moreover, we observed significant body weight loss in mice bearing *ADGRG6*-silenced tumors, suggesting possible systemic effects of *ADGRG6* inhibition. Although the underlying mechanism remains unclear, this phenomenon may involve altered cytokine production or host–tumor metabolic interactions, warranting further investigation into the systemic safety profile of targeting *ADGRG6*.

In summary, our findings establish *ADGRG6* as a multifaceted oncogenic regulator in PAAD that promotes tumor progression through both cell-autonomous mechanisms and potential immune-associated pathways. By integrating bioinformatic, experimental, and clinical evidence, this study expands the current understanding of *ADGRG6* biology in pancreatic cancer and provides a conceptual framework for exploring *ADGRG6* as a therapeutic target in future precision oncology and immunomodulation studies.

## 5. Conclusions

Based on the multifaceted evidence presented in this study, we conclude that *ADGRG6* is markedly overexpressed in PAAD and correlates with poor prognosis and immunoregulatory signaling. Through integrative multi-omics and functional validation, we demonstrate that *ADGRG6* promotes tumor proliferation, invasion, and migration, partly via activation of the NF-κB→STAT6→GATA3 axis. Although xenograft findings require validation in immunocompetent models, our results highlight *ADGRG6* as a potential oncogenic driver and therapeutic target in PAAD, linking tumor-intrinsic growth mechanisms with immune modulation within the TME.

## Figures and Tables

**Table 1:** Correlation between ADGRG6 and immune cell gene markers (TIMER and GEPIA).

Description	Gene Markers	Rho	*p*-Value	Rho	*p*-Value
		TIMER		GEPIA	
Mast Cells	*CPA3*	0.171	*	0.129	*
	*KIT*	0.285	***	0.28	***
M1 macrophage	*CD86*	0.148	*	0.15	*
	*CD80*	0.246	***	0.24	**
Neutrophils	*IFIT1*	0.199	**	0.21	**
	*RSAD2*	0.267	***	0.28	***
Th2 cell	*GATA3*	0.253	***	0.27	***
	*STAT6*	0.535	***	0.58	***
Th17	*STAT3*	0.37	***	0.39	***
	*BATF*	0.279	***	0.3	***

* *p* < 0.05, ** *p* < 0.01, *** *p* < 0.001. Neutrophils: Type I interferon response subgroup.

**Table 2:** Association of ADGRG6 expression with clinicopathological features in PAAD.

Clinicopathological Feature	Category	*n*	ADGRG6		*p* Value
			Low Expression (%)	High Expression (%)	
Gender	male	38	9 (23.68)	29 (76.32)	*p* = 0.153
	female	33	13 (39.4)	20 (60.6)	
Age	<60	23	6 (26.09)	17 (73.91)	*p* = 0.537
	≥60	48	16 (33.33)	32 (66.67)	
Tumor size	<3 cm	9	7 (77.78)	2 (22.22)	* *p* < 0.05
	≥3 cm	62	15 (24.2)	47 (75.8)	
Grade	I-II	40	19 (47.5)	21 (52.5)	*** *p* < 0.001
	III	31	3 (9.68)	28 (90.32)	
Nerve invasion	No	41	11 (28.95)	30 (71.05)	*p* = 0.376
	Yes	30	11 (22.92)	19 (77.08)	
Organ invasion	No	28	13 (46.43)	15 (53.57)	* *p* < 0.05
	Yes	43	9 (27.27)	34 (72.73)	
TNM stage	T1	3	3 (5.45)	0 (94.55)	** *p* < 0.01
	T2–3	68	19 (61.29)	49 (38.71)	
AJCC stage	1	13	7 (12.73)	6 (87.27)	* *p* < 0.05
	2–4	58	15 (48.39)	43 (51.61)	

*n* = 71, high expression = 49 (69.01%), low expression = 22 (30.99%). Statistical comparisons were conducted using the chi-square test. * *p* < 0.05, ** *p* < 0.01, *** *p* < 0.001.

## Data Availability

The original contributions presented in this study are included in the article and Supplementary Materials. Further inquiries can be directed to the corresponding author.

## Supplementary Materials

The following supporting information can be downloaded at: https://www.mdpi.com/article/10.3390/cimb47120991/s1.

## Abbreviations

Table following abbreviations are used in this manuscript:

ADGRG6	adhesion G-protein-coupled receptor G6
PAAD	pancreatic adenocarcinoma
TME	tumor microenvironment
TAMs	Tumor-associated macrophages
Tregs	Regulatory T cells
3D	Three-dimensional
siRNA, si-NC, si-ADGRG6	Small interfering RNA (and controls)
