# Supplementary material for: ADGRG6 Promotes Pancreatic Adenocarcinoma Progression Through the NF-κB/STAT6 Axis and Modulation of the Tumor Immune Microenvironment
Source: Curr Issues Mol Biol. 2025 Nov 27;47(12):991. doi: 10.3390/cimb47120991 (PMC12731683; doi:10.3390/cimb47120991)
Supplement: Supplementary file 1 [file cimb-47-00991-s001.zip › Table S2. Predicted dependency of pancreatic cancer cell lines on ADGRG6 (DepMap)..pdf]

Table S2. Predicted dependency of pancreatic cancer cell lines on ADGRG6 (DepMap).

Depmap ID	ADGRG6 Gene Effect (Chronos) CRISPR (DepMap Public 23Q4+Score, Chronos)	Primary Disease	Cell Line Name	Lineage
ACH-000022	0.176422235	Pancreatic Adenocarcinoma	PATU8988S	Pancreas
ACH-000023	-0.031310556	Pancreatic Adenocarcinoma	PATU8988T	Pancreas
ACH-000042	0.145304004	Pancreatic Adenocarcinoma	PANC0203	Pancreas
ACH-000060	0.119347161	Pancreatic Adenocarcinoma	PANC1005	Pancreas
ACH-000085	0.101964542	Pancreatic Adenocarcinoma	T3M4	Pancreas
ACH-000093	0.066071429	Pancreatic Adenocarcinoma	PANC0504	Pancreas
ACH-000094	0.100751988	Pancreatic Adenocarcinoma	HPAFII	Pancreas
ACH-000114	0.156565922	Pancreatic Adenocarcinoma	SU8686	Pancreas
ACH-000118	0.106105755	Pancreatic Adenocarcinoma	HUPT3	Pancreas
ACH-000138	0.165191902	Pancreatic Adenocarcinoma	CFPAC1	Pancreas
ACH-000139	-0.169154292	Pancreatic Adenocarcinoma	PANC0327	Pancreas
ACH-000155	0.184849697	Pancreatic Adenocarcinoma	SW1990	Pancreas
ACH-000164	0.08343874	Pancreatic Adenocarcinoma	PANC1	Pancreas
ACH-000178	0.183393723	Pancreatic Adenocarcinoma	HS766T	Pancreas
ACH-000213	0.088346149	Pancreatic Adenocarcinoma	HUPT4	Pancreas
ACH-000222	0.197062204	Pancreatic Adenocarcinoma	ASPC1	Pancreas
ACH-000235	0.153724326	Pancreatic Adenocarcinoma	PANC0403	Pancreas
ACH-000243	0.09589714	Pancreatic Adenocarcinoma	DANG	Pancreas
ACH-000265	-0.019734013	Pancreatic Adenocarcinoma	KP4	Pancreas
ACH-000266	0.109511103	Pancreatic Adenocarcinoma	SNU213	Pancreas
ACH-000281	0.023398344	Pancreatic Adenocarcinoma	KP2	Pancreas
ACH-000307	0.164514442	Pancreatic Adenocarcinoma	PK1	Pancreas

ACH-000320	0.102775399	Pancreatic Adenocarcinoma	PSN1	Pancreas
ACH-000332	0.144570307	Pancreatic Adenocarcinoma	YAPC	Pancreas
ACH-000354	0.200732315	Pancreatic Adenocarcinoma	CAPAN1	Pancreas
ACH-000417	0.082279575	Pancreatic Adenocarcinoma	PANC0813	Pancreas
ACH-000468	0.19555613	Pancreatic Adenocarcinoma	PK45H	Pancreas
ACH-000502	0.280098794	Pancreatic Adenocarcinoma	TCCPAN2	Pancreas
ACH-000517	0.123569813	Pancreatic Adenocarcinoma	SNU410	Pancreas
ACH-000535	0.145753922	Pancreatic Adenocarcinoma	BXPC3	Pancreas
ACH-000599	0.144865439	Pancreatic Adenocarcinoma	PATU8902	Pancreas
ACH-000601	0.129560633	Pancreatic Adenocarcinoma	MIAPACA2	Pancreas
ACH-000652	0.095645871	Pancreatic Adenocarcinoma	SUIT2	Pancreas
ACH-001098	0.260151087	Pancreatic Adenocarcinoma	KCIMOH1	Pancreas
ACH-001107	0.098133656	Pancreatic Adenocarcinoma	KP1N	Pancreas
ACH-001353	0.160723319	Pancreatic Adenocarcinoma	JOPACA1	Pancreas
ACH-001375	0.203924874	Pancreatic Adenocarcinoma	PACADD119	Pancreas
ACH-001377	0.083234206	Pancreatic Adenocarcinoma	PACADD137	Pancreas
ACH-001379	0.205503419	Pancreatic Adenocarcinoma	PACADD161	Pancreas
ACH-001380	0.166266371	Pancreatic Adenocarcinoma	PACADD165	Pancreas
ACH-001382	0.156280051	Pancreatic Adenocarcinoma	PACADD188	Pancreas
ACH-001999	0.111848339	Pancreatic Adenocarcinoma	9505BIK	Pancreas
ACH-002039	0.137172017	Pancreatic Adenocarcinoma	PK8	Pancreas
ACH-002186	0.200523922	Pancreatic Adenocarcinoma	PL4	Pancreas
ACH-002672	0.180428309	Pancreatic Adenocarcinoma	MAPACHS77	Pancreas
