# Supplementary material for: ADGRG6 Promotes Pancreatic Adenocarcinoma Progression Through the NF-κB/STAT6 Axis and Modulation of the Tumor Immune Microenvironment
Source: Curr Issues Mol Biol. 2025 Nov 27;47(12):991. doi: 10.3390/cimb47120991 (PMC12731683; doi:10.3390/cimb47120991)
Supplement: Supplementary file 1 [file cimb-47-00991-s001.zip › Table S3. Statistical comparisons of ADGRG6 mRNA expression across clinical subgroups in PAAD, based on TCGA data from the UALCAN database..pdf]

Table S3. Statistical comparisons of *ADGRG6* mRNA expression across clinical subgroups in PAAD, based on TCGA data from the UALCAN database.

Table S2A - Gender

Comparison	p-value	Significance
Normal-vs-Male	2.84280043949536E-10	***
Normal-vs-Female	1.98670000450818E-08	***
Male-vs-Female	6.099000E-01	ns

Table S2B - Pancreatitis Status

Comparison	p-value	Significance
Normal-vs-Pancreatitis	3.832100E-02	*
Normal-vs-NoPancreatitis	6.8121996932291E-10	***
Pancreatitis-vs-NoPancreatitis	2.555800E-01	ns

Table S2C - Age

Comparison	p-value	Significance
Normal-vs-Age(21-40Yrs)	4.473600E-01	ns
Normal-vs-Age(41-60Yrs)	1.30089999883509E-08	***
Normal-vs-Age(61-80Yrs)	6.31730001643405E-09	***
Normal-vs-Age(81-100Yrs)	3.891700E-02	*
Age(21-40Yrs)-vs-Age(41-60Yrs)	3.731200E-01	ns
Age(21-40Yrs)-vs-Age(61-80Yrs)	7.076400E-01	ns
Age(21-40Yrs)-vs-Age(81-100Yrs)	6.296000E-01	ns
Age(41-60Yrs)-vs-Age(61-80Yrs)	1.509600E-02	*
Age(41-60Yrs)-vs-Age(81-100Yrs)	4.638600E-01	ns
Age(61-80Yrs)-vs-Age(81-100Yrs)	5.324800E-01	ns

Table S2D – Drinking Habits

Comparison	p-value	Significance
Normal-vs-Non Drinker	1.339410E-03	**
Normal-vs-Daily Drinker	5.737800E-03	**
Normal-vs-Weekly Drinker	6.833900E-02	ns
Normal-vs-Occasional Drinker	1.022320E-03	**
Normal-vs-Social Drinker	2.741800E-03	**
Non Drinker-vs-Daily Drinker	1.696760E-01	ns

Non Drinker-vs-Weekly Drinker	5.677200E-01	ns
Non Drinker-vs-Occasional Drinker	3.485800E-01	ns
Non Drinker-vs-Social Drinker	2.604200E-01	ns
Daily Drinker-vs-Weekly Drinker	6.524800E-01	ns
Daily Drinker-vs-Occasional Drinker	5.542200E-01	ns
Daily Drinker-vs-Social Drinker	7.397800E-01	ns
Weekly Drinker-vs-Occasional Drinker	9.275200E-01	ns
Weekly Drinker-vs-Social Drinker	8.068800E-01	ns
Occasional Drinker-vs-Social Drinker	8.000800E-01	ns

Table S2E - Diabetes Status

Comparison	p-value	Significance
Normal-vs-Diabetic	6.154900E-04	***
Normal-vs-NonDiabetic	1.47299950015167E-10	***
Diabetic-vs-NonDiabetic	2.898000E-01	ns

Table S2F - Tumor Grade

Comparison	p-value	Significance
Normal-vs-Grade 1	2.522200E-01	ns
Normal-vs-Grade 2	9.221600E-02	ns
Normal-vs-Grade 3	2.251500E-02	*
Normal-vs-Grade 4	1.000000E+00	ns
Grade 1-vs-Grade 2	3.196200E-02	*
Grade 1-vs-Grade 3	7.980200E-03	**
Grade 1-vs-Grade 4	2.522200E-01	ns
Grade 2-vs-Grade 3	5.979200E-01	ns
Grade 2-vs-Grade 4	9.221600E-02	ns
Grade 3-vs-Grade 4	2.251500E-02	*

Table S2G - Nodal Metastasis Status

Comparison	p-value	Significance
Normal-vs-N0	1.600570E-04	***
Normal-vs-N1	4.29123403478116E-12	***
N0-vs-N1	9.349200E-01	ns

Table S2H - TP53 Mutation Status

Comparison	p-value	Significance
Normal-vs-TP53-Mutant	2.76120237785449E-11	***
Normal-vs-TP53-NonMutant	1.76039999999489E-06	***
TP53-Mutant-vs-TP53-NonMutant	1.751230E-01	ns
