# Supplementary material for: ADGRG6 Promotes Pancreatic Adenocarcinoma Progression Through the NF-κB/STAT6 Axis and Modulation of the Tumor Immune Microenvironment
Source: Curr Issues Mol Biol. 2025 Nov 27;47(12):991. doi: 10.3390/cimb47120991 (PMC12731683; doi:10.3390/cimb47120991)
Supplement: Supplementary file 1 [file cimb-47-00991-s001.zip › Table S5. Primer sequences for qRT-PCR detection of immune cell marker genes in tumor tissues from nude mouse xenograft models..pdf]

Table S5. Primer sequences for qRT-PCR detection of immune cell marker genes in tumor tissues from nude mouse xenograft models.

Gene Symbol	Forward Primer	Reverse Primer
cd68	GGACCCACAACTGTCACTCAT	AAGCCCCACTTTAGCTTTACC
ccr8	ACGTCACGATGACCGACTACT	CCCAGCACAAACAAGACGC
il13	CCTGGCTCTTGCTTGCCTT	GGTCTTGTGTGATGTTGCTCA
nrp1	GACAAATGTGGCGGGACCATA	TGGATTAGCCATTCACACTTCTC
gapdh	AGGTCGGTGTGAACGGATTTG	TGTAGACCATGTAGTTGAGGTCA
