# Supplementary figures and images for: ADGRG6 Promotes Pancreatic Adenocarcinoma Progression Through the NF-κB/STAT6 Axis and Modulation of the Tumor Immune Microenvironment
Source: Curr Issues Mol Biol. 2025 Nov 27;47(12):991. doi: 10.3390/cimb47120991 (PMC12731683; doi:10.3390/cimb47120991)

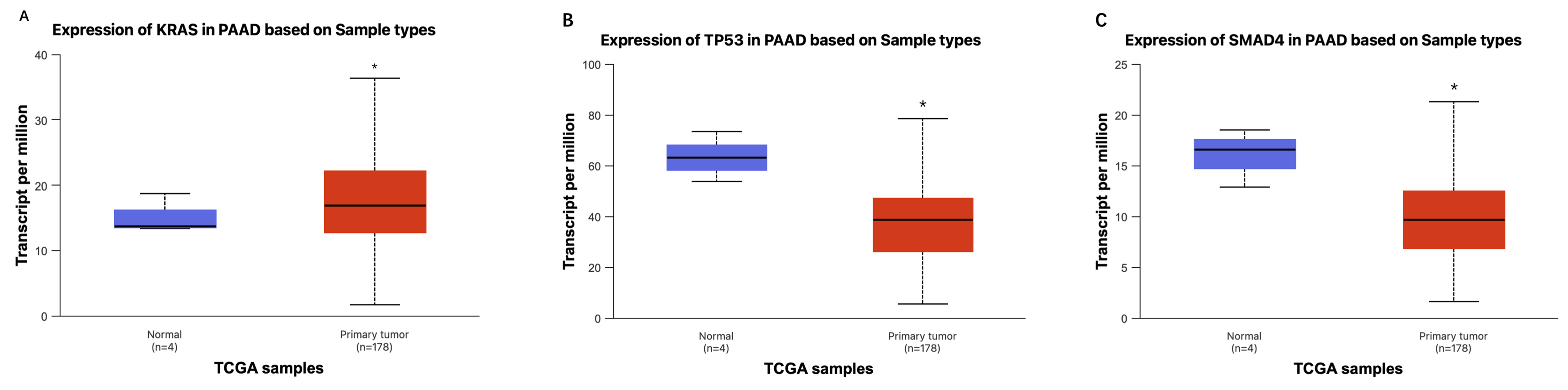

Supplement: Supplementary file 1 [file cimb-47-00991-s001.zip › Figure S1.tiff]

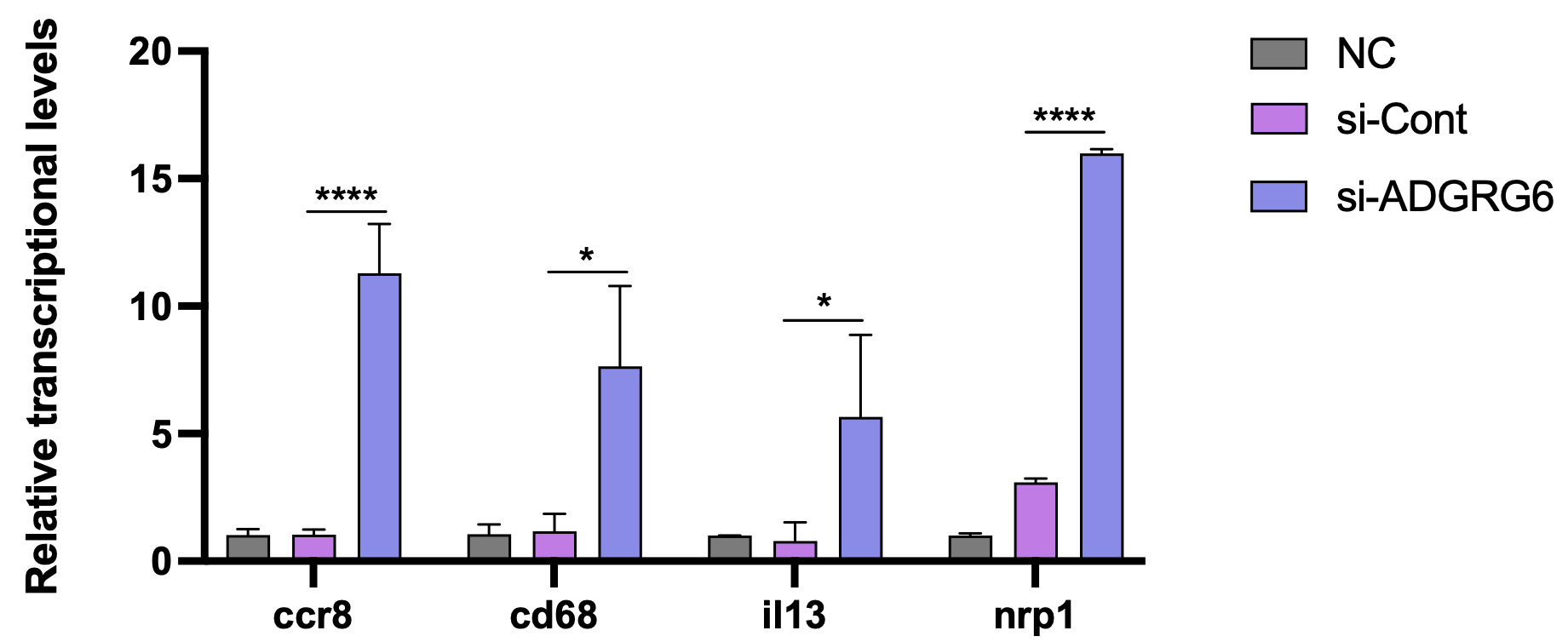

Supplement: Supplementary file 1 [file cimb-47-00991-s001.zip › Figure S3.tiff]
